# Airway and systemic biomarkers of health effects after short-term exposure to indoor ultrafine particles from cooking and candles – A randomized controlled double-blind crossover study among mild asthmatic subjects

**DOI:** 10.1186/s12989-023-00537-7

**Published:** 2023-07-10

**Authors:** Karin Rosenkilde Laursen, Nichlas Vous Christensen, Frans AA Mulder, Jörg Schullehner, Hans Jürgen Hoffmann, Annie Jensen, Peter Møller, Steffen Loft, Anna-Carin Olin, Berit B. Rasmussen, Bernadette Rosati, Bo Strandberg, Marianne Glasius, Merete Bilde, Torben Sigsgaard, The Climate Chamber Group

**Affiliations:** 1 Environment, Occupation and Health, Department of Public Health, Aarhus University, Aarhus, Denmark; 2 Interdisciplinary Nanoscience Centre (iNANO), Aarhus University, Aarhus, Denmark; 3 Department of Chemistry, Aarhus University, Aarhus, Denmark; 4 Geological Survey of Denmark and Greenland, Aarhus, Denmark; 5 Department of Respiratory Diseases and Allergy, Aarhus University Hospital, Aarhus, Denmark; 6 Section of Environmental Health, Department of Public Health, University of Copenhagen, Aarhus, Denmark; 7 Department of Public Health and Community Medicine, University of Gothenburg, Gothenburg, Sweden; 8 Faculty of Physics, University of Vienna, Vienna, Austria; 9 Division of Occupational and Environmental Medicine, Lund University, Lund, Sweden

**Keywords:** Indoor air, Ultrafine particles, Human exposure, Cooking, Candles, Inflammation, SP-A, Metabolomics, Biomarkers, Oxidatively damaged DNA

## Abstract

**Background:**

There is insufficient knowledge about the systemic health effects of exposure to fine (PM_2.5_) and ultrafine particles emitted from typical indoor sources, including cooking and candlelight burning. We examined whether short-term exposure to emissions from cooking and burning candles cause inflammatory changes in young individuals with mild asthma. Thirty-six non-smoking asthmatics participated in a randomized controlled double-blind crossover study attending three exposure sessions (mean PM_2.5_ µg/m^3^_;_ polycyclic aromatic hydrocarbons ng/m^3^): (a) air mixed with emissions from cooking (96.1; 1.1), (b) air mixed with emissions from candles (89.8; 10), and (c) clean filtered air (5.8; 1.0). Emissions were generated in an adjacent chamber and let into a full-scale exposure chamber where participants were exposed for five hours. Several biomarkers were assessed in relation to airway and systemic inflammatory changes; the primary outcomes of interest were surfactant Protein-A (SP-A) and albumin in droplets in exhaled air – novel biomarkers for changes in the surfactant composition of small airways. Secondary outcomes included cytokines in nasal lavage, cytokines, C-reactive protein (CRP), epithelial progenitor cells (EPCs), genotoxicity, gene expression related to DNA-repair, oxidative stress, and inflammation, as well as metabolites in blood. Samples were collected before exposure start, right after exposure and the next morning.

**Results:**

SP-A in droplets in exhaled air showed stable concentrations following candle exposure, while concentrations decreased following cooking and clean air exposure. Albumin in droplets in exhaled air increased following exposure to cooking and candles compared to clean air exposure, although not significant. Oxidatively damaged DNA and concentrations of some lipids and lipoproteins in the blood increased significantly following exposure to cooking. We found no or weak associations between cooking and candle exposure and systemic inflammation biomarkers including cytokines, CRP, and EPCs.

**Conclusions:**

Cooking and candle emissions induced effects on some of the examined health-related biomarkers, while no effect was observed in others; Oxidatively damaged DNA and concentrations of lipids and lipoproteins were increased in blood after exposure to cooking, while both cooking and candle emissions slightly affected the small airways including the primary outcomes SP-A and albumin. We found only weak associations between the exposures and systemic inflammatory biomarkers. Together, the results show the existence of mild inflammation following cooking and candle exposure.

**Supplementary Information:**

The online version contains supplementary material available at 10.1186/s12989-023-00537-7.

## Background

Indoor air quality is not well-regulated nor well-understood with respect to health effects. This knowledge gap is critical, as people spend up to 90% of their time indoors, and most of that time is spent in their home [1, 2]. Pollutants of indoor origin, such as dust, chemicals, and particulate matter (PM) are of great importance to personal exposure and, presumably health [3]. Numerous epidemiological studies have found high levels of PM in residences [4–10], with activities contributing to high levels of indoor particulate air pollution, including cooking and burning candles [4–7, 9]. Fine and ultrafine particles (UFP) including nanoparticles pose the greatest problems as they can penetrate deep into the lungs and enter the bloodstream thereby potentially affecting other organs including heart and brain [11–14].

Several studies have linked regular and prolonged exposure of indoor PM to adverse health especially in children, the elderly and asthmatics [15–17]. Among the wide range of organic compounds that are associated with PM, polycyclic aromatic hydrocarbons (PAHs), a large group of chemicals consisting of two to seven conjugated aromatic rings, are ubiquitous environmental contaminants. A single study found PAHs associated with lower lung function in asthmatic children [18]. Little is known about adverse health effects related to exposure to emissions from cooking and candle burning, as only a handful of studies assessing short-term health effects have been conducted. In the published studies on healthy subjects in exposure chambers with candle or cooking emissions, adverse effects on lung function [19], cardiovascular outcomes including blood pressure, arterial stiffness and heart rate variability [20–22], and brain activity [23, 24] have been demonstrated, though no single effect has been observed in all studies. In a previous publication from the present study, we have reported on changes in the nasal mucosa and exhaled nitric oxide (NO)-concentrations, the particle number and volume size distribution as well as the light scattering ability of the particles, and decreasing self-reported well-being following exposure to cooking and candle emissions among subjects with mild asthma [25]. In observational and intervention studies, indoor exposure to particles in the fine and ultrafine size range has been associated with systemic inflammatory biomarkers such as declining levels of endothelial progenitor cells, oxidative stress, and release of several cellular mediators, such as cytokines [26–29], all of above mechanisms relevant in the causal pathway to cardiovascular and pulmonary disease [29–31].

Lower airway responses to PM can be assessed by evaluating early biomarkers, including Surfactant Protein-A (SP-A) and albumin found in the lining fluid of small airways [32, 33]. SP-A poses several functions that make it an interesting potential biomarker for inflammation in the small airways [34]. Besides contributing to reduced surface tension in the alveoli during respiration, SP-A is a critical component of the respiratory innate immune defence; it is able to opsonize or bind pathogens and other invading micro-organisms to enhance phagocytic removal from the airways [35, 36]. It may also act as a modulator of the immune response [35].

Albumin, the most prominent blood protein, is the primary determinant for colloid osmotic pressure in the vascular space and possibly also in lining fluid of the small airways, but it is also suggested as a marker of membrane permeability [37]. Changes in concentrations of SP-A and albumin may indicate an inflammatory reaction [36]. Levels of SP-A and albumin are typically assessed by bronchoalveolar lavage (BAL) or similar invasive methods, but new technology makes it possible to measure these proteins in droplets in exhaled air [32, 33]. To date, the measurement of SP-A and albumin in exhaled air has not been studied in relation to exposure to air pollution, but effects on SP-A have been found in an exposure study of second-hand emissions from electronic cigarettes and in a cross-sectional study of smokers [38, 39].

Altered levels of serum metabolites, e.g. glycoprotein N-acetylation (GlycA) and cholesterol, may be associated with inflammation related to PM exposure [31, 40, 41]. Metabolomics offers valuable insight into the metabolic changes in response to low-dose PM exposure [31, 42], allowing the suggestion of hypotheses on mechanisms of toxicity in order to better understand the causes of diseases [42]. A recent intervention study showed associations of several serum metabolites with indoor fine particle exposure [31].

In the present study, the aim was to examine whether short-term respiratory and systemic effects of indoor exposure to cooking and candle emissions could be observed in a population of young asthmatic volunteers. Information on effects were collected in terms of SP-A and albumin in droplets in exhaled air, cytokines in nasal lavage, and cytokines, C-reactive protein (CRP), Epithelial Progenitor Cells (EPCs), genotoxicity, gene expression related to DNA-repair, oxidative stress, and inflammation, as well as metabolomics in blood. The hypothesis tested was that short-term exposure to cooking and candle emissions could induce acute responses in airways and blood. The PM properties are discussed in terms of estimated mass distributions and their hygroscopic properties are presented. For the convenience of the reader some description of exposure characteristics already described in [25] are summarized herein.

## Results

Results are presented as mean (± SD) unless specified otherwise.

### Particle exposure

The detailed characterization of exposure levels has been reported previously [25]. Due to air conditioning, temperature and relative humidity (RH) remained nearly constant throughout all exposures (~ 23 °C and ~ 43%). During candle exposure, levels of CO_2_ increased to 915 (± 66) ppm, and NO_2_ increased to 52.9 (± 1.8) ppb compared to CO_2_: 629 (± 74) and NO_2_: 2.1 (± 0.5) during clean air exposure. During cooking, levels of CO_2_ and NO_2_ were 542 (± 43) ppm and 6.5 (± 1.8) ppb, respectively.

**Fig. 1:**
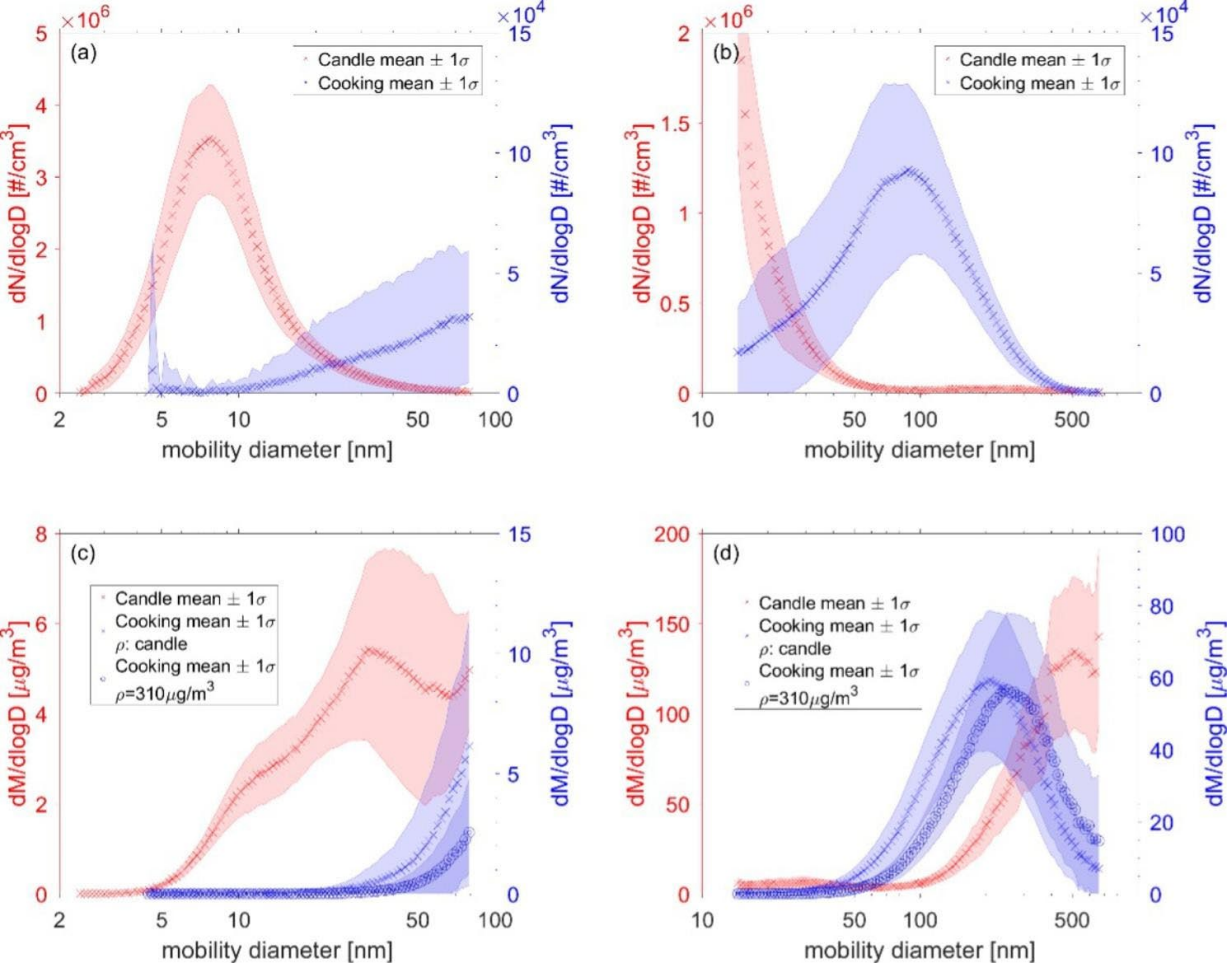
Mean particle size distributions recorded during cooking and candle exposure. **Legend**: Cooking in blue; (right y-axis) and candle exposures in red (left y-axis). Values show means as calculated from three cooking experiments carried out on 07.05.21, 07.11.19, and 30.10.19 and four candle experiments carried out on 09.05.19, 13.05.19, 21.11.19 and 05.11.19, respectively, using SMPS. See [25] for examples of size distributions from individual experiments. (**a**) Particle number size distributions in the range 2.4 to 79.1 nm (nano DMA). (**b**) Particle number size distributions in the range 14.6 to 661.2 nm (long DMA). (**c**) Particle mass size distributions in range 2.4 to 79.1 nm (nano DMA). (**d**) Particle mass size distributions in range 14.6 to 661.2 nm (long DMA). The two different SMPS size intervals were measured in sequence. The particle mass size distributions from cooking experiments are plotted assuming two different densities: blue crosses denote results calculated using the density assumed for candle emissions, while spheres denote results calculated using a density of 310 µg/m^3^

Representative examples of particle characteristics (number and volume size distributions and temporal evolution of the scattering coefficients) during a cooking, a candle, and a clean air exposure session are shown in our previous publication [25]. As a summary and for the convenience of the reader, mean particle number size distributions from three cooking and four candle exposures are shown in Fig. 1a and b demonstrating the difference in particle sizes in the two types of exposures. During cooking exposures, the particle mode diameter was 88 (± 20) nm (blue, Fig. 1a and b), while during candle exposure the mode diameter was 8 (± 1) nm (red, Fig. 1a and b). The mass of the particles and their water uptake potential (hygroscopicity), was also characterized. Assessing particle mass of combustion particles is challenging due to lack of information on particle density and morphology. Using two different densities to derive particle mass distributions, we found mode diameters of 209 (± 7) nm and 250 (± 70) nm for cooking exposures (see methods section). The actual density and thus mass size distribution of the particles from cooking exposure is unknown but expected to lie in-between the two blue lines (Fig. 1c and d). During candle exposure, a mode diameter of 514 (± 5) nm was found for the mean mass distribution. Interestingly, on the scale of the y-axis in Fig. 1c a distinct peak with a mode diameter of 32 (± 10) nm appears. As illustrated in Fig. 1d, the SMPS was not able to capture the full particle mass distribution, particularly for candle emissions, thus, we do not present total mass concentrations from this instrument.

**Fig. 2:**
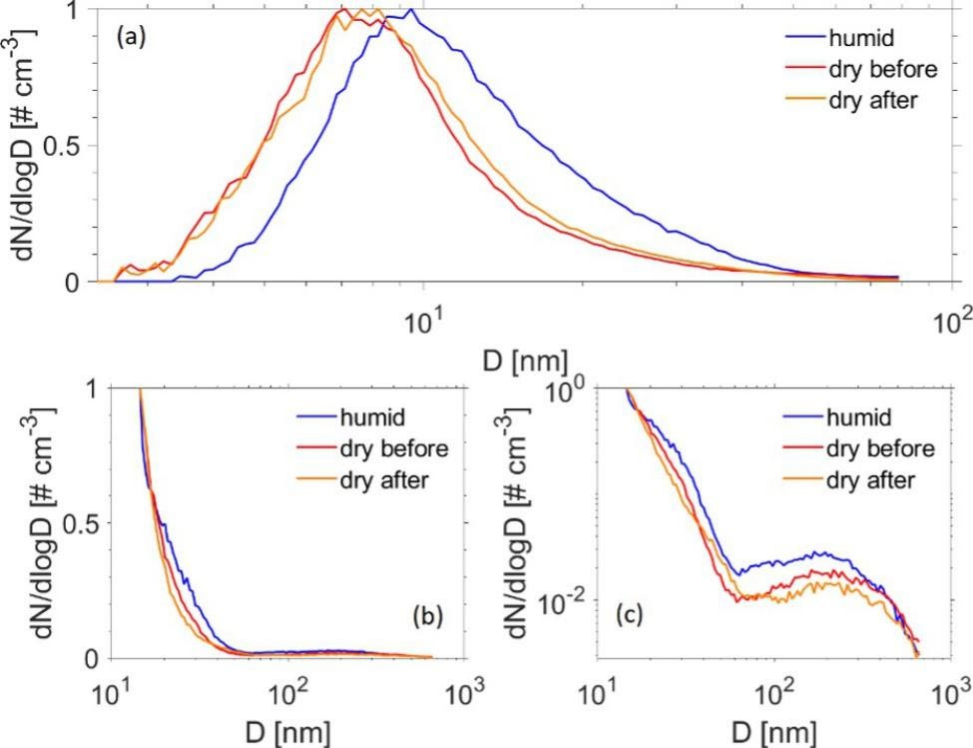
Normalized particle size distributions from candle experiments: **Legend**: (**a**) shows exposure session performed on 21.11.19 using nano DMA. (**b**) and (**c**) show exposure session performed on 05.11.19 using long DMA. Panels (b) and (c) show the same data but plotted with linear axis in (b) and logarithmic axis in (c) to better highlight the larger particle mode. Each curve was calculated as the median from 10 scans. The blue lines depicts the humidified distribution (RH ~ 90%), whereas the red and orange lines show the dry distributions recorded before and after the period of humidification (RH ~ 43%; conditions in the exposure chamber)

The hygroscopicity of cooking emissions was inconclusive, primarily due to the fact that the particle distributions varied strongly with time, as previously described in Laursen et al. [25]. Thus, subsequent measurements of dry and humid size distributions were difficult to interpret. Figure 2 illustrates candle emission size distributions before, during, and after humidification on two exposure days. Candle emissions in the size range 2.4 to 79.1 nm (nano DMA) showed some growth when exposed to high relative humidity: the mode diameter of the dry distribution, as calculated from the ten scans before and ten scans after humidification, shifted from 7.4 (± 0.2) nm to 9.5 (± 1.1) nm at humid conditions. The RH in the humidifier was set to 90%, but a slightly lower RH is expected inside the SMPS. To account for particle loss inside the setup (including the humidifier) we performed calculations with the Particle Loss Calculator [43] that suggest that less than 10% of particles with a diameter of 5 nm would be additionally lost in the expanded setup with a humidifier compared to measurements of the dry distributions. Thus, we ascribe the shift to larger sizes, illustrated in Fig. 2, to be mainly due to the growth of the particles by water uptake rather than loss of small particles in the system. Candle emissions in the larger size ranges did not seem to exert the same hygroscopic growth as observed in the smaller size ranges (Fig. 2b and c), suggesting a different chemical composition.

### Scanning electron microscope images

In terms of SEM images, we were not able to visually distinguish the cooking filter sample from the reference filter, thereby not able to see particles from the cooking exposure session maybe due to evaporation during the analysis. The fibers from the filter itself can be seen clearly in both a) and c).

**Fig. 3:**
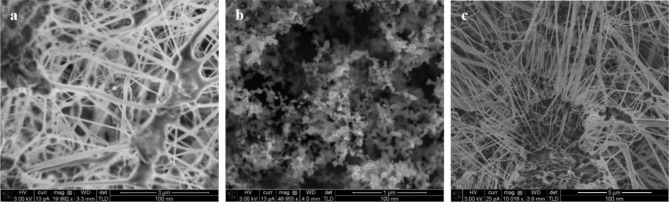
Scanning Electron Microscope (SEM) images of filters from exposures sessions. **Legend: a**) a cooking exposure, **b**) a candle exposure and **c**) an unused reference filter. From b) candle particles and agglomerates down to 20 nm can be observed

### Polycyclic aromatic hydrocarbons

The results (average and range) for all 16 US EPA PAHs are given in Table S5. The range for sum PAHs for the three exposure scenarios was clean air 0.16–1.3 (average 1.0) ng/m^3^, cooking 0.88–1.6 (average 1.1) ng/m^3^ and candle burning 7.8–21 (average 10) ng/m^3^. There was a small but still clear trend that all PAH groups were slightly higher in the cooking sessions compared to clean air. Significantly higher levels in the candle experiments were measured by a factor of ten compared with clean air and cooking. Thus, the results show that both cooking and candle burning are sources of PAH. The elevated PAH levels in the candle experiments on the PM_2.5_ filters consisted mainly of 2–3 ring PAHs, which are PAHs mainly found (> 90%) in the gas phase.

### Biomarkers

Tables 1 and 2 present the estimated changes following cooking and candle exposures for the included respiratory and systemic biomarkers, respectively. The magnitude of effects is reported in terms of coefficients from linear mixed-effects models on datasets normal scale or transformed data using log- or cube root transformation. The strength of associations can be inferred by the size of 95% confidence intervals (95% CI), whereas effect size cannot be compared across biomarkers because of differences in scales. Table 3 presents changes in metabolites and macromolecules. In Tables S1 and S2, unadjusted means and standard deviations for the included biomarkers can be found.

**Table 1:** Mean change in respiratory outcomes following cooking and candle exposure (clean air = reference)†

		Cooking exposure			Candle exposure		
		Coefficient	95% CI	*p*-value	Coefficient	95% CI	*p*-value
**Biomarkers in exhaled air**							
	SP-A %	0.02	(-0.30; 0.35)	0.888	0.31	(-0.02; 0.63)	0.065
	Albumin %	0.24	(-0.26; 0.74)	0.343	0.25	(-0.25; 0.75)	0.325
	Albumin/SP-A	0.08	(-0.10; 0.25)	0.243	-0.05	(-0.22; 0.13)	0.591
**Nasal lavage biomarkers**							
	IL-1β	-0.20	(-0.40; -0.01)	0.044^*^	-0.09	(-0.29; 0.11)	0.370
	IL-8	-0.05	(-0.23; 0.14)	0.634	-0.03	(-0.21;0.16)	0.777

Mean changes for nasal lavage biomarkers correspond to differences on logarithmic scale

^†^ Results are from linear mixed models with no interaction. Changes in biomarkers in exhaled air are reported from 5 to 24 h post exposure adjusted for baseline (0 h). For nasal lavage biomarkers no baseline values exist. SP-A and albumin are expressed as weight%. IL-1β and IL-8 are reported in pg/ml. *Definition of abbreviations*: SP-A = Surfactant Protein-A, IL = interleukin. * The level of significance was assumed at *p* < 0.05

**Table 2:** Mean change in systemic inflammation biomarkers following cooking and candle exposure (clean air = reference)†

		Cooking exposure			Candle exposure		
		Coefficient	95% CI	*p*-value	Coefficient	95% CI	*p*-value
**Cytokines in serum**							
	IL-1β	-0.14	(-0.31; 0.02)	0.086	-0.17	(-0.32; -0.01)	0.036^*^
	IL-8	0.14	(-0.70; 0.97)	0.743	0.18	(-0.63;0.99)	0.660
	CCL2	3.19	(-11.1; 17.5)	0.660	18.3	(3.97; 32.7)	0.013^*^
	TNF-α	-0.42	(-0.78; -0.06)	0.023^*^	-0.54	(-0.91; -0.17)	0.004^*^
**C-Reactive Protein**							
	CRP	0.14	(0.03; 0.25)	0.010^*^	0.10	-0.01; 0.20	0.075
**EPCs**							
	Early	0.74	(-55.7; 57.2)	0.979	-4.55	(-61.7; 52.6)	0.875
	Late	-2.55	(-33.7; 28.6)	0.871	-9.08	(-41.8; 23.7)	0.585
**Gene expression**							
	*IL-8*	0.22	(-0.20; 0.63)	0.313	0.39	(-0.03; 0.80)	0.068
	*CCL2*	-0.13	(-0.54; 0.29)	0.554	0.01	(-0.41; 0.42)	0.983
	*TNF-α*	-0.10	(-0.53; 0.33)	0.637	-0.04	(-0.46; 0.39)	0.855
	*HMOX1*	-0.06	(-0.37; 0.25)	0.700	0.01	(-0.30; 0.32)	0.966
	*OGG1*	-0.20	(-0.51; 0.09)	0.175	-0.07	(-0.36; 0.22)	0.645
**DNA damage**							
	Strand breaks	-0.007	(-0.04; 0.02)	0.677	0.002	(-0.03; 0.03)	0.918
	Fpg-sensitive sites	0.06	(0.01; 0.11)	0.024^*^	-0.02	(-0.07; 0.04)	0.556

Mean changes for CRP and Gene expression correspond to differences on logarithmic scale. Mean changes for DNA damages correspond to differences on the cube root scale

^†^ Results are from linear mixed models with no interaction term. Changes are reported from 5 to 24 h post exposure adjusted for baseline (0 h). For cytokines, only CCL2 had complete data; for IL-1β: 167/324, IL-8: 207/324, and TNF-α: 204/324 observations were included in the analyses. *Definition of abbreviations*: CCL2 = C-C motif chemokine ligand 2, EPCs = Endothelial Progenitor Cells. Fpg = formamidopyrimidine DNA glycosylase. HMOX1 = heme oxygenase (decycling) 1, IL = interleukin, TNF-α = tumor necrosis factor α, OGG1 = oxoguanine DNA glycosylase 1. Cytokines in serum are reported in ln(pg/ml). CRP is reported in ln(ng/ml). EPCs are reported in number of endothelial cells per standard unit. DNA-damages are reported in lesions per 10^6^ base pairs. * The level of significance was assumed at *p* < 0.05

**Table 3:** Mean change in metabolites and macromolecules on days with cooking exposure compared to clean air exposure†

	Cooking exposure			
Metabolite / macromolecule	Chemical shift (ppm)	Cooking#24 hours	95% CI	*p*-value
Unsaturated fatty acid = CH	~ 5.25	-43.50	(-82.37; -4.63)	0.028
Unsaturated fatty acid = CH-CH_2_	~ 2.04	94.31	(20.83; 167.80)	0.012
Unsaturated fatty acid = CH-CH_2_	~ 2.04	106.02	(30.20; 181.85)	0.006
Unsaturated fatty acid = CH-CH_2_	~ 2.04	135.28	(15.57; 255.00)	0.027
Unsaturated fatty acid = CH-CH_2_	~ 1.97	99.31	(12.31; 186.30)	0.025
Alanine	~ 1.45	120.47	(32.22; 208.71)	0.008
Unidentified	~ 1.45	99.21	(12.72; 185.70)	0.025
Unidentified	~ 1.45	95.51	(9.81; 181.20)	0.029
Unidentified	~ 1.45	94.54	(9.27; 179.81)	0.030
Lipid -CH3 (+ Valine)	~ 1.00	107.89	(15.07; 200.71)	0.023
Lipid -CH3 (+ Valine)	~ 0.94	144.14	(14.50; 272.78)	0.028

^†^ Metabolites and macromolecules are shown if (*p* ≤ 0.03). Results are derived from linear mixed models of Nuclear Magnetic Resonance (NMR) data using Model 1 with interaction between exposure and time. *Definition of abbreviations*: ppm = parts per million

**Fig. 4:**
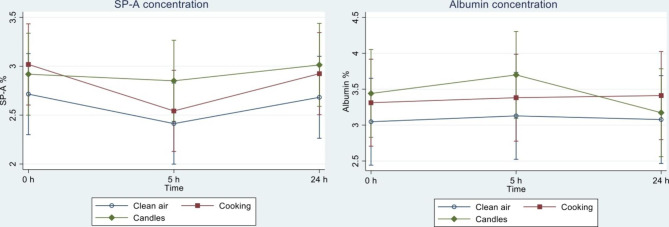
Margins plot of the adjusted means in biomarkers in exhaled air **Legend**: For each of the three exposures clean air, cooking, and candles Surfactant Protein-A and albumin were measured before exposure (0 h), and following exposure corresponding to 5 h after and 24 h after exposure start as depicted on the x-axis. SP-A and albumin are reported in % of the sampled material

*SP-A and albumin in exhaled air*: Fig. 4 illustrates the adjusted mean concentrations of SP-A and albumin for the three exposures over time. The figure shows that the concentration of SP-A was approximately constant over time when participants were exposed to candle emissions, while it tended to decrease five hours after exposure start following clean air and cooking exposure. Compared to days with clean air exposure, mixed models showed that concentrations of SP-A in the samples were higher following candle exposure (0.31% (95% CI -0.02; 0.63)) (Table 1). The difference between candle and clean air exposure on SP-A was persistent across analyses, but with varying significance (Table 1, S3 and S4). There was no difference between cooking and clean air exposure on SP-A when observing changes following the exposures adjusted for baseline values (Table 1). Exposure to cooking and candles was associated with higher concentrations of albumin in samples compared to clean air exposure; cooking: 0.24% (95% CI -0.26; 0.74) and candles: 0.25% (95% CI -0.25; 0.75). The difference in albumin concentration was persistent across analyses, although none were statistically significant (Tables S4 and S5). The effect size of the albumin/SP-A ratio was 0.08 (95% CI -0.10; 0.25) for cooking and -0.05 (95% CI -0.22; 0.13) for candles.

*Nasal lavage biomarkers*: We observed a significant decrease in IL-1β from 5 to 24 h following cooking exposure (-0.20 (95% -0.40; -0.01)), but no clear change in IL-1β following candle exposure (-0.09 (95% CI -0.29; 0.11)) compared to clean air exposure (Table 1). No significant differences between the exposures were observed for IL-8.

*Cytokines in serum*: Several of the measurements were below the lower detection limit. This was true for IL-1β, IL-8, and TNF-α, and missing data were excluded from the analyses. The results of the remaining cytokines are presented in Table 2. IL-1β and TNF-α showed a significant or near-significant decline from 5 to 24 h following cooking and candle exposure compared to clean air exposure (Table 2). No significant association between the exposures and IL-8 was observed. CCL2 increased significantly from 5 to 24 h following candle exposure compared to clean air: 18.3 pg/ml (95% CI 3.97; 32.7). There was a significant difference in CCL2 changes following candle vs. cooking exposure, with candles increasing levels of CCL2 significantly more than cooking: 15.2 pg/ml (95% CI 1.12; 29.2) (data not shown).

*C-reactive protein (CRP) in serum*: The concentration of CRP in plasma was not increased following exposure to emissions from candles or cooking. Estimates in Table 2 indicate increasing CRP following both exposures; however, the margins plot showed an actual decline in CRP following clean air exposure and approximately stable levels for the particle exposures.

*Endothelial progenitor cells*: Table 2 shows EPC levels stratified by phenotypes, early and late. No significant effect of cooking and candle exposure was observed for neither early nor late EPCs. Linear mixed models supplemented with Student’s t-test for changes over time for each exposure showed significant and borderline significant increases for early and late EPCs between 0 and 5 h for all exposures suggesting diurnal effects (data not shown). Sensitivity analyses of samples stratified by dilution showed no significant associations between the exposures and EPCs (data not shown).

*Gene expression in peripheral blood mononuclear cells (PBMCs)*: The measured gene expression in PBMCs related to DNA repair and pro-inflammatory responses did not show any significant variations following cooking or candles exposure (Table 2), except from a borderline significant positive regression coefficient of *IL-8* following candle exposure (0.39 (95% CI: -0.03; 0.80)). Analyses showed significant variations in time for *HMOX1*, *OGG1*, and *TNF-α* following all exposures with increasing values from 0 to 5 h (data not shown).

*DNA damage in PBMCs*: Cooking and candle exposure had no significant effect on the level of DNA strand breaks; however, elevated levels of Fpg-sensitive sites were observed following cooking exposure compared to following clean air exposure (regression coefficient on cube root scale = 0.06 (95% CI: 0.01; 0.11). As seen from Table 2, no difference in levels of Fpg-sensitive sites was observed when comparing candle exposure to clean air exposure.

*Metabolomics*: From the analysis of Nuclear Magnetic Resonance (NMR) spectroscopy data (see Figure S1 for example spectra and S2 for Quality Control) several significant peaks in metabolites and macromolecules were observed. In particular, we observed increasing concentrations of lipids and lipoproteins following cooking exposure compared to when participants were exposed to clean air (Table 3). Peaks around ~ 2 ppm correspond to glycoprotein N-acetylation (GlycA) [41]; however, due to the untargeted metabolomics approach, it was not possible to specify macromolecules further. No significant associations were found for metabolites following candle-burning exposure.

## Discussion

To our knowledge, this study including the companion paper by Laursen et al. 2021 [25] represents the first controlled human exposure study of the impact of cooking and candle-burning exposure in subjects with mild asthma. We found suggestive evidence that five-hour exposure to emissions from cooking and candles, respectively (at PM_2.5_ mass concentrations ~ 90 µg/m^3^), slightly changed the primary outcome measures, SP-A and albumin in droplets in exhaled air, with SP-A affected differently by candles compared to cooking and clean air, and albumin increasing numerically following exposure to cooking and candles, although not significantly. Cooking exposure was associated with elevated levels of oxidatively damaged DNA measured by Fpg-modified comet assay and increased concentrations of some lipids and lipoproteins in the blood. Only weak, no, or reducing effects were observed for other secondary outcomes in terms of the upper airway and systemic inflammatory biomarkers, EPC levels and gene expression. Serum CRP decreased following clean air exposure.

As shown in Laursen et al. 2021 [25], during cooking exposure, participants were exposed to a lower number of particles compared to when exposed to candle emissions. Our previous publication shows that the mode diameter (number size distribution) during cooking exposure is much larger compared to that measured during candle exposure, similar to findings in the literature with candles emitting high number concentrations of UFP with a diameter < 10 nm [44–46], and soot particles having a mean diameter of ~ 270 nm [22, 47]. When, however, looking into the particle mass distributions, the major mode diameter measured during candle exposure exceeds the one from the cooking exposure. This can be explained by a second peak measured during candle exposure that appears at sizes between 100 and 500 nm that is however much lower in number and thus not visible in Fig. 1b. This second peak is though visible when plotting the number size distributions on a log-log scale as seen in Fig. 2c. As the mass of the particles is dependent on the third power of the particle size, the role of the large particles is strongly enhanced. For candle particles, we observed different behavior of the size ranges regarding water uptake, with the smaller particles showing more growth than the larger particles when exposed to high humidity. The relatively high hygroscopicity for the small particles is consistent with earlier findings by Li and Hopke, who, however, did not measure the larger particle fraction [48]. Previous candle emission studies showed that especially the small particle sizes contain considerable amounts of salts, while soot particles govern the larger size ranges [47, 49]. While salts are very hygroscopic, soot particles are known to be more hydrophobic, which might explain the difference in particle growth behavior with size.

The concentrations of PAHs in the present study are low compared to reported levels in indoor and outdoor air all over Europe [50]. However, the levels in our study are quite similar compared to a Swedish study, that reported benzo(a)pyrene levels between 0.011 and 0.14 ng/m^3^ indoors [51]. Also, the results from our study are similar to the highest values reported for PAHs on PM_2.5_ samples in a study on emissions of particles and gases from stressed burning of five types of pillar candles with different wax and wick composition [52]. Differences between the cooking and candle exposures regarding particle size and chemical composition of the emissions might explain the difference observed in health effects [14].

As explained in Laursen et al. 2021 [25] we aimed for the same mass concentration level for the two types of exposures (cooking and candle) and across sessions and obtained average particle mass concentrations (PM_2.5_) of 96.1 (± 13.1) µg/m^3^ and 89.8 (± 9.3) µg/m^3^ from filter samples for cooking and candles, respectively. These values are higher compared to the mass concentrations calculated from the SMPS data. The differences most likely arise due to the different size ranges chosen and in the case of cooking emissions selected densities that might not reflect those of the actual cooking aerosols.

Lower airway effects were assessed by evaluating novel and early biomarkers from the distal part of the lungs [32, 33]. SP-A and albumin are abundant proteins in the lung lining fluid, forming an interface between lung epithelial cells and the external environment [53]. In the present study, we observed different effects on SP-A concentrations following the three exposures, with differences between the candle and clean air exposure on SP-A concentrations being significant or borderline significant across statistical analyses. We were not able to establish whether the difference in effects are caused by a decreasing effect of clean air or an increasing effect of candle emissions on SP-A; however, stable levels during candle exposure and recent research on diurnal variation in healthy non-exposed individuals, showing minor increases in SP-A during the day, point to a decreasing effect of clean air exposure on SP-A [54]. This may be explained by an increase in respiration rates as a consequence of the particle-free clean air, hence, a greater use of surfactant with the small airways opening and closing more frequently. Decreasing levels of NO in exhaled air has been shown in healthy individuals following particle-free clean air exposure [55], indicating that the airways may be subject to small inflammatory effects during everyday life as a consequence of continuous, minor exposure to pollutants. This may be especially true for individuals with asthma [56]. The cause of the observed decrease in SP-A following cooking exposure is unknown. It may be explained by changes in the lung milieu [34]; however, further studies are needed.

Damage to the small airways may increase the permeability of the blood-air space barrier, leading to the passage of plasma proteins into the airway space and possible leakage of lung proteins out from the airways. This in turn, may change the protein content [57]. Inflammation is generally associated with albumin leakage from the vasculature into the airways [58, 59] – a possible explanation for the observed tendencies towards increasing albumin concentrations in the small airways following cooking and candle exposure. When albumin increase in the small airways, interstitial osmotic pressure may be increased [37].

In an exploratory approach in the present study, we found increased levels of several lipoproteins following cooking exposure – a metabolic change, which is commonly observed following inflammation due to increased apolipoprotein synthesis [60]. For all reported metabolites, parallel increases were observed following candle exposure; however, they were not as pronounced as for cooking and not significant at *p* ≤ 0.03. Had we chosen a significance level < 0.05, more than 100 significant peaks in metabolites were found for cooking exposure when compared to clean air, possibly indicating further metabolic changes. However, with ~ 1000 bins tested, many of these were likely to be false positives. In a randomized blinded intervention study using air purifiers in dormitories among healthy young adults, high PM_2.5_ exposure was likewise associated with alterations in serum lipid metabolites, indicating an enhancement of lipid metabolism and oxidation [31]. The changes in lipids and lipoproteins that occur during inflammation are part of the innate immune response and, therefore likely to play an important role in protecting the host [60, 61]. Evidence shows, that acute inflammation and infection induce various alterations in lipid metabolism, but if the inflammatory response persists, it may contribute to an increased risk of atherosclerosis [60]. We found that significant changes in peaks of unsaturated fatty acids following cooking exposure corresponded to GlycA [41, 61], which may be consistent with inflammation. Results from recent observational and interventional studies have demonstrated that GlycA is elevated in acute and chronic inflammation, suggesting GlycA being a marker that tracks systemic inflammation and subclinical vascular inflammation [41, 61]. Previous results have suggested that GlycA captures systemic inflammation at least as well as CRP [41, 61, 62]. GlycA is a composite biomarker integrating protein levels and glycosylation states of the most abundant acute phase proteins in serum, allowing for a stable measure of inflammation [61].

Exposure to cooking was associated with elevated levels of oxidatively damaged DNA, normally occurring when oxidative stress and inflammation are present [63]. Previous studies show that exposure to combustion particles is consistently associated with oxidatively damaged DNA in humans [64, 65]. Replication of damaged DNA may lead to structural changes or mutations to the chromosomes, events which are critical in the development of cancer [65, 66]. Furthermore, an increased level of DNA damage caused by oxidative reactions represents a relevant event in the pathway leading to chronic disease and eventually also to death [65, 66]. Candle exposure did not affect DNA damage measured by strand breaks or lesions detected as Fpg-sensitive sites in DNA. This is in line with earlier observations on intra-tracheal instillation of candle burning particles that showed unaltered levels of genotoxicity, although the exposure caused both pulmonary inflammation and increased protein content in BAL fluid [67]. Differences between the effect of cooking and candle exposure may be explained by different physicochemical properties of particles and by different compounds emitted, such as PAHs in the gas phase, which often constitutes > 90% of total and VOCs as these have previously been shown to play important roles in the oxidative potential of particles [68].

In general, low concentrations of the different cytokines in nasal lavage fluid were observed. We found slightly lower levels of interleukins after cooking exposure, significant for IL-1β. Previous studies show a clear downward shift in all cytokines and cells concentrations from the first nasal lavage to the subsequent ones [69]. In order to avoid this, we refrained from sampling at baseline since this might have induced artificially lower levels after exposure.

In several studies, enhanced levels of serum cytokines have been used to determine the systemic inflammation level in humans exposed to air pollution [11]. Overall, no evident effects in systemic biomarkers (EPCs, gene expression, CRP, and cytokines) were found in the present study. Though, following candle exposure, we observed a significant increase in circulating CCL2 (from 5 to 24 h) indicating continuous and increasing inflammation from baseline. CCL2 recruits cells of the immune system (monocytes, lymphocytes etc.) to the sites of inflammation produced by tissue injury or infection [70]. Inhaled particles can provoke an inflammatory response in the lungs, with consequent release of inflammatory cytokines into circulation – typically including interleukins and TNF-α [11, 71]. We found borderline significant increases in gene expression related to *IL-8* following candle exposure. In contrast, we observed very small; however, decreasing levels of *IL-1β* and *TNF-α* following cooking and candle exposure compared to clean air exposure. This might indicate recruitment from the blood of these cytokines into the cell lining as a first response [69]. Analyses showed significant variations in time for *HMOX1*, *OGG1*, and *TNF-α* following all exposures with increasing values from before exposure (morning) to after exposure (afternoon). This effect may be related to the stay in the exposure chamber or diurnal variation, which is similar to the findings of a recent study on gene expressions in humans after controlled exposure to a hydrogenated vegetable oil exhaust [72]. There was a slight increase in some serum cytokines following clean air exposure, which is probably an effect caused by the stay in the exposure chamber. Despite the fact that the exposure order was randomized, baseline values between exposures clearly deviated from each other for some of the outcomes. In the statistical analyses, we therefore adjusted for baseline values. We have no reasonable explanation for this variation other than a low number of participants. By using a randomized cross-over design and by preparing participants before taking part in the study, we did everything possible to prevent this variation.

Systemic inflammation may be observed by elevated CRP, as found in several cross-sectional studies among children and healthy adults [73]; however, in the present study, cooking and candle exposure did not alter CRP levels in serum. CRP decreased following clean air exposure, particularly at 24 h, which might be explained as an effect of very clean air in the chambers during the clean air sessions compared to standard indoor and ambient air. The air delivered to the chambers were filtered through a series of filters, including a final stage with HEPA- and carbon filters. Similarly, in a recent intervention study, air filtration was associated with decreased concentrations of inflammatory markers including CRP [74].

### Strengths and limitations

As explained in Laursen et al. 2021 [25], particular strengths of the present study were the design, including randomization, and double blinding. Combining this design with a state-of-the-art exposure chamber, in which all conditions other than the exposures were kept constant, eliminates confounding from personal characteristics. In general, controlled human exposure studies, make it possible to separate the effects of the specific PM component and size fraction of different combustion sources from effects associated with the complex mixtures of air pollution examined in epidemiological studies [75, 76]. As confirmed by an “exit poll” among the participants on their final visit, as described previously [25], blinding of candle exposure proved successful, strengthening the results. Contrary, we were only able to blind cooking to investigators, not participants, because of the smell of roasted pork. Nevertheless, it is unlikely that participants’ knowledge about the exposure affected the objective measures reported here. In brief, the exit poll was completed the morning after participant’s third exposure. On a paper, participants marked which exposure they thought they had been exposed to on their day one, two and three, respectively. Comparing their actual exposure to their appraised exposure made it possible to evaluate the participant blinding effectiveness of the study. On exposure days with cooking, 35/36 (97.2%) participants were able to identify the exposure. Participants were not able to identify whether they had been exposed to clean air or candles in a systematic way; when exposed to candles 20/35 (57.1%) participants guessed the exposure correctly. A Chi^2^-test showed no significant difference (p = 0.250) whether participants thought they had been exposed to candles or clean air on days with candle exposure and vice versa. All participants have been exposed to the same concentration of particles and gases, as exposure levels were constant throughout and across exposure days. In the present study, particle levels from candles and cooking are comparable to real-life scenarios [4, 6, 8, 77]. A particular strength of our study is the thorough exposure characterization performed with several instruments, including SMPS giving particle size and number concentrations down to 2.4 nm. For candles in particular, this is important, as evidence, including findings from the present study, indicates high number of concentrations of particles below 10 nm [44, 45]. We examined a comprehensive array of biomarkers previously associated with air pollution and from several places in the human body, providing a thorough understanding of how individuals may be affected by indoor particles.

The present study also has limitations. First, exposure to indoor and ambient pollution between the days of the experiments might impact the results, as participants were left unattended in their homes with no instructions regarding behaviour except for not using tobacco products and not taking medicine. However, due to the crossover design and randomization of the exposures, the activities of participants in the hours and days before the exposure sessions are expected to cause random effects, thereby attenuating the exposure-outcome association. Secondly, in the case of delayed effects, the health effects of cooking and candle burning may have been underestimated in the present study. However, as the exposures are not assumed to be receptor-mediated as, e.g. endotoxin showing systemic effects persisting for weeks [78], we do not expect a cascade of inflammation, but instead, general mild inflammation to occur – which might, however, not decrease – within a short amount of time [65, 79]. Thirdly, the clinical outcomes might have changed differently, if we had examined candles composed of other materials and/or under other burning conditions. Other cooking styles e.g. cooking on a stove most likely would have emitted different profiles of compounds and different levels of PM [80] affecting deposition in the respiratory tract and consequently health reactions [81]. Yet, the examined exposures were chosen as being representative of Denmark and other Nordic countries. Fourth, in order to generate similar exposure scenarios across study exposure days, there were some differences to real-life exposure patterns in a common household. In order to reduce uneven emissions from soot and burning fat, we replaced candles before burning down, and pork was kept in the turned-off oven when finished. However, not opening the ovens most likely reduced contamination with combustion particles and PAHs compared to real-life scenarios. Fifth, due to the sampling protocol used in this study, the exposure and health risk was estimated without considering the gaseous PAH fraction. Finally, as individuals with asthma are particularly vulnerable to particle exposure due to chronic inflammation in the respiratory tract, the findings indicating mild inflammatory responses do not necessarily pertain to the general population. Nevertheless, the results may apply to susceptible individuals such as children, the elderly, and other individuals with chronic respiratory disease – also known to be susceptible to PM exposure [82, 83]. However, as several of our key biomarkers showing possible effects of the exposures (biomarkers in exhaled air as well as GlycA and other lipid metabolites) are new, but promising in relation to air pollution, the interpretation of actual health effects is difficult. Furthermore, secondary outcomes needs to be viewed upon as exploratory, and hence, warranting further investigation.

## Conclusions

In conclusion, the results of this study suggest that emissions from cooking and candle burning can affect parts of the respiratory system thereby causing a shift in some local and systemic biomarkers in young individuals with asthma, thus, possibly pointing to the existence of mild inflammation. Cooking and candle burning induced different effects on health, which may be explained by differences in particle size and chemical composition of the emissions. As key findings in the present study are related to novel biomarkers, the findings warrant confirmation in future studies. Nevertheless, strategies to reduce indoor particle pollution should be considered to minimize potential disease progression.

## Methods

Details on study design, participants, exposure facilities, exposure generation, and exposure characterization have been described elsewhere [25] and are summarized below.

### Study design

In short, the study was designed as a randomized, double-blind, controlled crossover exposure experiment. Participants took part in three exposure sessions, each lasting five hours; (a) air mixed with emissions from cooking (mean fine particle mass concentration (± SD)) (PM_2.5_: 96.1 (± 13.1) µg/m^3^), (b) air mixed with emissions from burning candles (PM_2.5_: 89.8 (± 9.3) µg/m^3^), and (c) clean filtered air (PM_2.5_: 5.8 (± 6.8) µg/m^3^). The filtered clean air and particle sessions were identical except for the air quality. Participants were exposed in groups of four, with each participant attending all three exposure sessions, with a gap of two weeks between each exposure.

### Study population

Thirty-six non-smoking individuals (20 female; 16 male) with mild asthma participated in the study (mean age (± SD): 22.3 (± 1.5) years) [25]. Participants had to be without signs of infections or airway symptoms and not have taken steroids for at least one week or any medicine during the least 48 h before participating in an exposure session. This was affirmed during a medical check-up in the morning before each exposure session.

### Exposure facilities and exposure description

Exposure sessions took place in a 72.9 m^3^ exposure chamber made of stainless steel, while exposure generation took place in a similar, but smaller adjacent chamber. Because of an established negative pressure of 10 Pa in the large exposure chamber, particles and gases were directed from the adjoining chamber to the large exposure chamber through a 10 m pipe connection. On days with cooking as exposure, four ovens were placed in the adjoining chamber. One oven at a time was cooking breast of pork (28% fat) at 200 °C as prescribed on the packaging. Before the first oven finished cooking the meat, the next oven started, and so forth, until the first oven had to start over again with new meat. In total, the four ovens cooked meat five times in order for the exposure to last throughout the exposure session. On exposure days with burning candles, four taper candles and six pillar candles made of 100% stearin were lit and placed on a table. In the chamber, a light circulation of air was made by a wide slow-rotating fan, which made the candles flicker at a slow pace. A big funnel was placed above the table, absorbing emissions from the candles, thereby transferring them into the exposure chamber, where it was mixed with a constant inflow of clean air. During clean air sessions, the adjacent chamber was not in use. In order to maintain a stable exposure level, different average air exchange rates were applied for the three exposures; (average air exchange (± SD) during cooking: 4.4 h^− 1^ (± 0.2); candles: 3.5 h^− 1^ (± 0.1); clean air: 2.6 h^− 1^ (± 0.4)). Throughout exposure sessions, the target temperature was 23 °C and relative humidity 45% inside the large exposure chamber. Before the first participant in the group of four entered the exposure chamber, the exposure had been activated for approximately two hours to ensure that the particle concentration had reached the required target concentration. Participants entered the exposure chamber with 30 min in between starting their five-hour exposure session. During exposure, participants were seated around a desk in a resting position wearing clean-suits to avoid unintended contamination of the air from clothes etc.

### Data collection

#### Exposure characterization

The particle exposure inside the exposure chamber was monitored and characterized during each exposure session from the first person entering the chamber until the last person leaving the chamber. Online monitoring of particle mass was performed by a Dusttrak Aerosol Monitor 8520 equipped with a PM2.5 inlet (TSI, St Paul, Minnesota) to control the exposure level. Particles (PM_10_ and PM_2.5_) were sampled using SKC PTFE filters with PMP Support by means of PM-samplers (SKC PEM 2.5 μm, 2 L/min, and ADI PM 2.5 μm & PM 10 μm, 10 L/min). Particle size distributions were measured at several exposure sessions using a Scanning Mobility Particle sizer (SMPS) equipped with a nano Differential Mobility Analyzer (DMA) (TSI, 3085) (nano DMA, particle size range 2.4–79.1 nm) or a long DMA (TSI, 3081) (long DMA, particle size range 14.6-661.2 nm). The two size intervals were measured in sequence during an exposure session. In order to calculate the mass size distributions from number size distributions retrieved with the SMPS system for candle emissions, we assumed an effective density and mass mobility exponent according to Rissler et al. [84] and Andersen et al. [52] as found from direct mass mobility measurements for candle soot. For particles smaller than 32 nm in diameter, a constant density of 1500 kg/m^3^ was chosen as proposed by Park et al. [85] and Pagels et al. [47], while the density of larger particles (> 32 nm) gradually decreased from 1500 kg/m^3^ to approximately 100 kg/m^3^. As no parametrizations exist for cooking aerosols, we assessed their density by comparing PM_2.5_ gravimetric results with measurements from DustTrak, finding an average density of 310 µg/m^3^. The SMPS results showed that the majority of particles from cooking have a diameter far below 2.5 μm.; hence, we also chose to calculate the mass size distributions with the density published for particles from candle burning. The density from candle burning represents a lower limit, while the PM_2.5_ gravimetric results from the DustTrak represents an upper limit, which is why the actual density of the cooking particles is expected to lie somewhere in between. During the experiments performed on 05.11.2019 and 21.11.2019, a humidifier was placed in front of the SMPS for a period of approximately half an hour to measure the particle size distribution after exposure to a relative humidity of 90 (± 2) % at the inlet of the SMPS. By comparing the size distributions with and without a humidifier, the hygroscopic growth of the poly-disperse particle distribution could be addressed.

Images of SKC PTFE filters (PM_2.5_) from cooking and candle sessions and a reference filter were taken using scanning electron microscopy (JEOL Magellan XHR 400 FE-SEM 3 kV nominal current 13pA spot size ~ 1-1.5 nm). Filter samples were imaged without any added conducting coating to prevent changes to the sample materials. It was not possible to apply higher magnification or longer exposure times of filters in the microscopy as this could lead to beam-induced damage of the filter material.

The Supplementary section thoroughly describes the analytical method and quality assurance results for the PAH analysis. In brief, before extraction of the PM_2.5_ filter samples, an internal standard mixture containing the 16 US EPA priority PAHs were added to the samples. The target compounds were analyzed using an Agilent GC/MS/MS system 7010B GC/TQ coupled to an 8890 GC system (Agilent Technologies).

#### Clinical measurements and biomarkers

Prior to (0 h), right after (5 h), and the morning after (24 h) exposure, each participant underwent several health examinations, including a sampling of exhaled air, nasal lavage, and blood. For all outcomes, participants served as their own controls. All clinical investigations were timed, so that they were performed at approximately the same time of the day before and after each exposure session. Proteins in droplets in exhaled air, comprising surfactant protein-A and albumin, were the primary outcome of interest in the study. Other outcomes reported in this study are secondary outcomes of interests, which is why they have to be viewed as hypothesis-generating. The effect of cooking and candle exposure on respiratory markers of inflammation and self-reported well-being has been reported elsewhere [25].

*SP-A and albumin in exhaled air*: Droplets in exhaled air, also termed particles in exhaled air, were collected using the PExA® instrument set-up [32, 33], which is a non-invasive method to assess the lining fluid from the distal airways [86]. Endogenous particles, formed in the airways, are exhaled and reflect the chemical composition of the respiratory tract lining fluid [32]. Participants performed repeated breath maneuvers allowing for airway closure and re-opening as described previously [57]. The subjects exhaled through a mouthpiece and a two-way, non-rebreathing valve into the thermostated PExA instrument (36 °C), containing a Grimm 1.108 optical particle counter and an impactor with a Teflon membrane impaction substrate. Participants inhaled HEPA-filtered air for three breaths before the sampling in order to remove particles originating from ambient air. Participants wore a nose clip throughout the procedure. They were instructed to perform the following standardized breathing maneuvers to allow for airway closure and re-opening: (i) exhale fully to residual volume and hold breath for five seconds, (ii) inhale rapidly to total lung capacity, (iii) exhale to residual volume capacity at a flow of 1000–1500 mL/s. The exhalation flow was shown to the participant on a computer screen. Only the exhalation in (iii) was sampled in the instrument. The maneuver was repeated until 120 ng was collected or a maximum sampling time of 30 min was reached, with normal tidal breathing in between. After collection, the Teflon membrane was immediately transferred to a low-binding Eppendorf polypropylene vial and stored at -80 °C until analysis [87]. Samples were analyzed for SP-A and albumin using mass spectrometry. Details on the instrument and analysis have been described elsewhere [57]. Four of 324 samples were excluded from the statistical analyses, as they were contaminated with saliva, detected by extremely high levels of albumin. Results are reported as weight%, herein % of the sampled material.

*Cytokines in nasal lavage:* Participants sat with a fully flexed neck when sampling nasal lavage. Through a nasal cork plug attached to a syringe, 5 mL of 0.9% sterile saline water (~ 37 °C) was injected into one nostril. The saline water was kept in the nasopharyngeal region for 30 s, followed by a collection of the fluid in a cup. The lavage was then repeated in the other nostril. The first nasal lavage sample (flush from the right and left nostril) was collected after exposure (5 h) and then again at follow-up (24 h). No baseline sample was performed to avoid “cleaning” the nasal cavity prior to exposure. Each nasal lavage sample was transferred to a vial, 30mM Dithiothreitol was added with the amount of fluid determined by differential weighing, and the sample was separated into a pellet and the supernatant. The supernatant samples were kept on ice during processing (approximately 15 min), following centrifuge (10 min at 755 g and 4 °C). Supernatant samples (2 × 1 mL per sample) were stored in cryo-tubes at -80 °C until analysis. The supernatant samples were analyzed for interleukin-1β (IL-1β) and interleukin-8 (IL-8) using Magnetic Luminex Performance assay (R&D Systems, Minneapolis, MN). 100 µL undiluted sample and 25 µL of a suspension of capture-antibody-conjugated beads were mixed in plate wells. After three hours of incubation, the beads were washed three times and subsequently reacted for 1.5 h with a 50 µL mixture of biotin antibody cocktail detection antibodies. 50 µL of streptavidin-phycoerythrin was added to the wells, and the incubation was continued for an additional 30 min. Finally, the beads were washed three times and re-suspended in 100 µL buffer, and analyzed on the Luminex® MAGPIX platform using xMAP technology. All samples were measured in duplicate. Results are reported in pg/ml.

*Blood samples*: Four mL of peripheral venous blood was sampled in K_2_-tubes (BD Vacutainer®, Denmark) containing EDTA as an anticoagulant for endothelial progenitor cells (EPC). Next, 8 mL blood used for measurement of gene expression was collected in CPT vials (BD Vacutainer®, Denmark). Finally, for analyses of cytokines, CRP and metabolomics, 10 mL blood was sampled in SST advance tubes (BD Vacutainer®, Denmark). A Safety-Lok™ blood collection set (BD Vacutainer®, Denmark) was applied. Following gradient centrifugation, the peripheral blood mononuclear cells (PBMCs) for measurement of gene expression were stored at -80 °C in a freezing medium containing 50% fetal bovine serum (GibcoRBL), 40% RPMI-1640 medium, and 10% dimethyl sulfoxide. Samples for cytokines, CRP, and metabolomics were stored at room temperature for 20 min before centrifuged (15 min at 755 g and 4 °C). Serum blood was transferred to three 1.8 mL microtubes (Sarstedt, Nümbrecht) before being stored at -80 °C until analysis. The samples underwent different procedures, as described below.

*Cytokines in serum*: After thawing, serum samples (2 × 1.8 mL) were analyzed using Magnetic Luminex® Performance assay (R&D Systems, Minneapolis, MN). A portion of a 50 µL standard undiluted sample and a 50 µL diluted microparticle cocktail were mixed in plate wells. After three hours of incubation on a microplate shaker (800 rpm, room temperature), samples were washed three times using a magnetic device for microplates. 50 µL Biotin-Antibody Cocktail was added to each well following incubation for one hour in a microplate shaker (800 rpm). Subsequently, samples were washed three times. Streptavidin-phycoerythrin (50 µL) was added to each well, and the incubation was continued for an additional 30 min. Finally, the beads were washed and resuspended in 100 µL wash buffer, following incubation for two minutes at room temperature on a microplate shaker (800 rpm). Within 90 min the samples were analyzed on the Luminex® MAGPIX platform using xMAP technology. All samples were measured in duplicate. The concentration was measured for Tumor Necrosis Factor-α (TNF-α), C-C motif chemokine ligand 2 (CCL2), IL-1β, and IL-8. Results are reported in pg/mL.

*C-reactive protein (CRP)*: Serum samples were analysed using Quantikine® ELIZA kit, Human C-Reactive protein (R&D Systems, Minneapolis, MN). 50 µL undiluted sample was diluted 1:50 − 1:400, dependent on CRP levels in the sample. 50 µL standard and diluted samples and 100 µL of Assay diluent were mixed in plate wells, following incubation for two hours at room temperature. Subsequently, samples were washed four times. 200 µL of Human CRP Conjugate was added to each plate well, then incubated for two hours and washed once. 200 µL substrate solution was added to plate wells and incubated for 30 min while protected from light. 50 µL stop solution was added to each well. The optical density of each well was determined within 30 min, using a microplate reader set to 450 nm (Walvelenght correction was set to 570 nm) using GENS software. All samples were measured in duplicate. Results are reported in ng/ml.

*Endothelial Progenitor Cells (EPCs)*: Fresh EDTA blood from the participants (4 mL) was analyzed 24 h after exposure start. Thus, blood from before, 5 h after, and 24 h after exposure start was analyzed at the same time for one exposure session. The blood had been stored at 5ºC until analyses. The collected blood samples were analyzed for EPCs using polychromatic flow-cytometry, defining EPCs as events within the leukocyte gate with a CD34^+^KDR^+^ antigenic profile expressed as percent EPCs per leukocyte, as described by Jantzen et al. [88]. We further used the presence or absence of the differential progenitor marker CD133^+/−^ to separate the EPCs into early or late subpopulations, respectively, as the surface marker CD133 expressed in EPCs upon release into circulation is lost upon maturation allowing discrimination between early or late EPCs [89]. Blood samples (1 mL) from the study participants were hemolysed with Ammonium Chloride buffer at RT in the dark for 20 min and centrifuged (10 min at 400 g). The supernatant was discarded, and the remaining 100 µL cells were stained with CD133 BV480 (1 µL, BD Catalog NR 747,562), CD34 PerCP Cy5.5 (20 µL, BD Catalog No. 347,222) and CD309 PE (20 µL, BD Catalog no. 560,494) and 30 µL Brilliant Violet binding buffer (BD Catalog no. 563,794) in a master mix (15 min, 25ºC, dark). The samples were diluted to 2 mL and aliquoted in 500 µL onto a 98-well deep-well plate. The samples were acquired at 500 µL/minute with an Attune Flow Cytometer from Thermo Fisher with a threshold set on violet Forward Scatter. Leukocytes were gated on a SS:FS scatter plot, and CD34 + cells were gated on a SS:CD34 plot. CD309^+^ cells were divided into CD133^+^ (early) and CD133^−^ cells (late), avoiding neutrophil background. With an Attune Flow Cytometer, all cells in 1 mL blood were processed, and equivalent fractions of the samples was compared. For the first 36 of 324 samples, the dilution factor was double (4 mL). Accordingly, these were analyzed separately in a sensitivity analysis. Results are reported in a number of endothelial cells per standard unit (1 mL).

*Gene expression in PBMCs*: The expression of the genes related to DNA repair (oxoguanine DNA glycosylase 1 (*OGG1*), GenBank sequence accession ID: 4968)) and oxidative stress (heme oxygenase (decycling) 1 (*HMOX1*), Gene ID: 3262), as well as genes related to inflammation interleukin 8 (*IL-8*, Gene ID: 3576), *TNF-α* (Gene ID: 7124), and chemokine (C-C motif) ligand 2 (*CCL2*, Gene ID: 6347) were analyzed in PBMCs. Total RNA was isolated using a Direct-zolTM RNA MiniPrep kit (Zymo Research, Irvine, CA, USA), which included a DNase I treatment. The PBMCs diluted in a freezing medium was centrifuged (10 min at 400 g and 4 °C), and the TRI Reagent was added to the precipitate, as stated in the protocol for biological liquids. The quantitative PCR reactions were carried out in ABI PRISM 7900HT (Applied Biosystems), using probes and primers from Applied Biosystems. The assay IDs for the genes were as follows: CCL2, Hs00234140_m1; IL6, Hs00985641_m1; IL8, s00174103_m1; TNF, Hs00174128_m1; HMOX1, Hs00157965_m1; OGG1, Hs01114116_gl. The 18 S rRNA was used as a reference gene (Eukaryotic 18 S rRNA Endogenous Control, 4352930E, Applied Biosystems). The PCR reactions were performed as described by Jensen et al. [90]. The level of gene expression is reported as the ratio between the level of the target gene and the 18 S rRNA reference gene using the comparative 2^−ΔCt^ method.

*DNA damages in PBMCs*: Levels of DNA strand breaks and oxidatively damaged DNA were determined by the alkaline comet assay as previously described and reported according to the Minimum Information for Reporting on the Comet Assay (MIRCA) recommendations [91, 92]. The oxidatively damaged DNA was analysed using the lesion-specific bacterial repair enzyme formamidopyrimidine DNA glycosylase (Fpg). PBMC suspensions (75 µL) were mixed with 600 µL of 0.75% agarose gel, and 120 µL of this suspension was applied onto Gelbond films (Cambrex, Medinova Scientific A/S, Hellerup, Denmark). The gel-embedded cells were lysed overnight (2.5 M NaCl, 100 mM Na_2_EDTA, 10 mM Trizma base). Sixty microliters Fpg (1 mg/mL, NorGenoTech, Norway) or enzyme buffer (40 mM HEPES, 0.1 M KCl, 0.5 mM Na_2_EDTA, 0.2 mg/mL bovine serum albumin) were applied onto the gels and covered with a coverslip. The slides were subsequently incubated at 37 °C for 45 min in a moist box in an incubator. The samples were afterward placed in electrophoresis buffer (1 mM Na_2_EDTA, 300 mM NaOH) for 40 min, and the electrophoresis was subsequently run for 25 min at 300 mA and 20 V (0.83 V/cm; cathode to anode). The samples were placed in a neutralization buffer (0.4 M Trizma base) for 15 min, followed by 90 min treatment in 96% ethanol to preserve the embedded samples. The nuclei were stained with YOYO^TM^-1 dye (491/509; Thermo Fisher Scientific, Waltham, MA, USA) and scored manually under an Olympus CX40 fluorescence microscope at 40x magnification. The samples were blinded when scoring the comets, and the DNA damage level was determined using a five-class scoring system (arbitrary score range of 0-400). For each sample, 100 randomly chosen nucleoids per slide were visually scored. The level of Fpg-sensitive sites was calculated as the difference in the score from samples incubated with Fpg and buffer. THP-1 cells exposed to 2.5 mM potassium bromate (KBrO_3_) was used as positive controls for oxidatively damaged DNA, as KBrO_3_ generates high levels of oxidatively damaged DNA and low levels of DNA strand breaks [93, 94]. THP-1 cells exposed to 50 µM H_2_O_2_ (1.13 ± 0.14 lesions/10^6^ base pairs (bp), n = 36) were used as positive controls for DNA strand breaks, as H_2_O_2_ generates high levels of DNA strand breaks and low levels of oxidatively damaged DNA [95]. Based on 36 independent assays, the means and standard deviations of the assays controls were as follows: DNA strand breaks = 0.07 ± 0.05 lesions/10^6^ bp, (0 µM H_2_O_2_) and 1.13 ± 0.14 lesions/10^6^ bp, (40 µM H_2_O_2_); Fpg-sensitive sites = 0.03 ± 0.02 lesions/10^6^ bp (0 mM KBrO_3_) and 0.97 ± 0.10 lesions/10^6^ bp (2.5 mM KBrO_3_). The comet score was transformed to lesions per 10^6^ bp using an investigator-specific calibration curve, where one arbitrary unit (0-400 arbitrary unit scale) corresponds to 0.00574 /10^6^ bp as described previously [96].

*Metabolomics*: NMR spectroscopy was used for metabolomics. Frozen serum blood samples were thawed, and 1 mL was transferred to SampleJet NMR tubes (Bruker®, Karlsruhe, Germany). A small amount of paramagnetic gadoteridol ‘relaxation agent’ was added in order to guarantee the quantitative response of the NMR spectrometer [97] to a final concentration of 0.3 mM. NMR samples received at the NMR facility were kept at 6 °C. Time to experimentation varied from 4 to 24 h as samples were automatically taken from 96-tube racks in succession. NMR analyses were done on a Bruker 500 MHz spectrometer equipped with a SampleJet automatic sample changer, using 5 mm sample tubes. All measurements were done at 310 K (37 °C), and automation was run from the Bruker IconNMR module. In order to ease the comparison of intensities of all spectra, the autogain option (rga) was disabled, and all experiments were recorded with oversampling and a receiver gain of 90.5. All samples placed in the SampleJet were kept cooled at 6 °C. Drying and heating was done for 60 s before loading samples into the magnet core to prevent condensed air on the tubes. Once the sample was positioned, the temperature was equilibrated for 120 s until the temperature stability was better than 0.2 K, followed by automatic shimming and tune/match. The time spent on each sample change totaled 5 min. Each 1D proton spectrum measurement (experiment NOESYGPPR1D) consisted of 4 dummy scans and 96 scans, with 1 s relaxation delay between scans and 1 s for signal acquisition. The total acquisition time per sample was three and a half minutes. 1D-NOESY NMR spectra were pre-processed in parallel in TopSpin 4.0.9, with a small line broadening of 0.3 Hz, a phase correction, water peak removal, and a spline-corrected baseline correction. Following the pre-processing, all data was gathered in a matrix using *nmrglue* [98] prior to spectral alignment with *Icoshift* [99]. The alignment was performed with an initial co-shift of 0.004 ppm following a squared average alignment of manually defined bins surrounding the critical areas in the spectra. Furthermore, all spectra were referenced to the glucose peak at 5.22 ppm. Following the alignment, all spectra were binned and integrated into two regions, namely from 9.60 ppm to 5.16 ppm and 4.30 ppm to -0.500 ppm, in bin sizes of 25 points (≈ 0.009 ppm), giving rise to a total of 1007 bins, which were Pareto scaled for each sample. With that, the dataset of 1007 variables for each sample were used in further statistical analysis as described below.

### Statistics

We used linear mixed models based on the univariate repeated measurement analysis of variance (ANOVA) to evaluate the change in health outcomes between clean air and candles and cooking, respectively. The models included the outcome of interest and as fixed effects exposure, time, exposure-order, day, and time-exposure interaction. As a random effect, we included participant ID. Time was divided into baseline (0 h), five hours, and 24 h; the exposure was clean air, candles, or cooking; order corresponding to the order in which the participant received the exposure, while day indicated whether the exposure took place on participants’ first, second or third day. The statistical measures of interest were the exposure, and time-exposure interaction as an effect of any of these terms would indicate a difference associated with the exposure. We initially fitted a model with interaction (Model 1). For models where the interaction term was not statistically significant, the interaction term was left out. Instead, we examined the mean change in the outcomes following the three exposures (5 to 24 h) adjusted for baseline values (0 h) (Model 2). In case of non-normal distributions, analyses were performed on log-transformed outcome variables. This was true for cytokines in nasal lavage, serum CRP, and gene expression. For DNA damages, outcomes were analysed on a cube root scale. Before conducting the statistical analyses on the 1007 metabolomics bins, we decided to use a false-discovery rate of *p* ≤ 0.03 to keep spurious findings low but still enable explorative analyses. For other outcomes, the significance level was assumed at *p* < 0.05. All statistical analyses were performed using Stata 17 software (StataCorp, College Station, Tex).

## Electronic supplementary material

Below is the link to the electronic supplementary material.

Supplementary Material 1

## Data Availability

The data used and analysed during the current study are available from the corresponding author on reasonable request.

## Abbreviations

CCL2: C-C motif chemokine ligand 2
CRP: C-reactive protein
CO_2_: carbon dioxide
EPC: endothelial progenitor cells
Fpg: formamidopyrimidine DNA glycosylase
GlycA: glycoprotein N-acetylation
HMOX1: heme oxygenase (decycling) 1 gene
IL-8: interleukin-8
IL-1β: interleukin-1β
ng: nanogram
nm: nanometer
NMR: Nuclear Magnetic Resonance
NO_2_: nitrogen dioxide
OGG1: oxoguanine DNA glycosylase 1
PAH: Polycyclic Aromatic Hydrocarbons
PExA: Particles in Exhaled Air
pg: picogram
PM: particulate matter
ppb: parts per billion
ppm: parts per million
SEM: Scanning Electron Microscope
SP-A: Surfactant Protein A
TNF-α: Tumor necrosis factor-α
UFP: ultrafine particles
µg: microgram
µL: microliters
