# Supplementary material for: Airway and systemic biomarkers of health effects after short-term exposure to indoor ultrafine particles from cooking and candles – A randomized controlled double-blind crossover study among mild asthmatic subjects
Source: Part Fibre Toxicol. 2023 Jul 10;20:26. doi: 10.1186/s12989-023-00537-7 (PMC10332087; doi:10.1186/s12989-023-00537-7)
Supplement: Supplementary file 1 — Supplementary Material 1 [file 12989_2023_537_MOESM1_ESM.docx]

**__________________________________________________________________________**

**SUPPLEMENTARY INFORMATION**___________________________________________________________________________

**Airway and systemic inflammation biomarkers after short-term exposure to indoor ultrafine particles – A randomized controlled double-blind crossover study among mild asthmatic subjects**

Karin Rosenkilde Laursen^1^, The Climate Chamber Group^1^, Nichlas Vous Christensen^2,3^, Frans AA Mulder^2,3^, Jörg Schullehner^1,4^, Hans Jürgen Hoffmann^5^, Annie Jensen^6^, Peter Møller^6^, Steffen Loft^6^, Anna-Carin Olin^7^, Berit B. Rasmussen^3^, Bernadette Rosati^3,8^, Bo Strandberg^9^, Marianne Glasius^3^, Merete Bilde^3^, Torben Sigsgaard^1^

^1^ Environment, Occupation and Health, Department of Public Health, Aarhus University, Denmark. ^2^ Interdisciplinary Nanoscience Centre (iNANO), Aarhus University, Denmark. ^3^ Department of Chemistry, Aarhus University, Denmark. ^4^ Geological Survey of Denmark and Greenland, Aarhus, Denmark. ^5^ Department of Respiratory Diseases and Allergy, Aarhus University Hospital, Denmark. ^6^ Section of Environmental Health, Department of Public Health, University of Copenhagen, Denmark. ^7^ Department of Public Health and Community Medicine, University of Gothenburg, Sweden. ^8^ Faculty of Physics, University of Vienna, Austria. ^9^ Division of Occupational and Environmental Medicine, Lund University, Sweden.

**Analysis of Polycyclic aromatic hydrocarbons (PAHs)**

*Chemicals and Reagents*All adsorbents, silica gel 60 (Merck, Darmstadt, Germany) and sodium sulfate (Merck, Darmstadt, Germany) were cleaned by thermal treatment at 450°C and activated at 100°C before use. All solvents were of glass distilled quality (Merck, Darmstadt, Germany). A deuterated internal standard mixture (1 ng µL^-1^) containing the 16 U.S. Environmental Protection Agency (US-EPA) priority PAHs (Dr. Ehrenstorfer (Augsburg, Germany) were used. Native mixture at 1 ng L^-1^, containing the 16 US EPA PAHs (Dr. Ehrenstorfer, Augsburg, Germany), were used for detection and quantification of target compounds. Octachlorornaphthalene (OCN) (Ultra Scientific, North Kingstown, RI, USA) (1 ng L^-1^) was used as recovery standard (RS).

*Sample extraction, clean-up and analysis*The samples (PM_2.5_ filter) were placed in glass vials and spiked with a portion of 40 μL of the internal standard mixture, and then extracted by sonication for 15 min in 3 mL of dichloromethane at maximal amplitude in a Sonica Ultrasonic Extractor (Soltec, Milan, Italy). Following extraction, the samples were cleaned up using a Pasteur pipet, with a small plug of wool in the bottom, filled with 2 cm silica powder and some sodium sulfate on top. The elute was evaporated under nitrogen flow until only a third of the initial volumes was left, solvent exchanged using n-hexane (~3 mL), and finally evaporated to ca 200 µL. Samples were transferred to GC glass inserts vials (Agilent Technologies) and 40 µL of RS was added and samples were reduced to a small volume (~30-40 µL) for analysis.
Table S6 shows all investigated compounds. Target compounds were separated on an Agilent GC/MS/MS system 7010B GC/TQ coupled to an 8890 GC system (Agilent Technologies). Samples (0.2 μL) were injected using an Agilent autosampler unit 7693A. The capillary column used was a DB-5MS (30 m × 0.25 mm, 0.25 μm, Agilent Technologies). Helium was the carrier gas at a flow rate of 1.0 mL/min. The temperature program was as follows: initial temperature 50°C for 3 min; ramp at 10°C/min to 180°C and held for 5 min; ramp at 3°C/min to 300°C and held for 10 min; injection at oven temperature at 250°C; and transfer line at 250°C. Electron impact ionization (EI) was performed at 70 eV energy at a 280°C ion source temperature. Scan type used was MRM/SIM filtering.

*Quality assurance*

Consistent recoveries (40-110%) were obtained for all IS compounds that were added to and used for correction of the samples. Field and laboratory blanks were analyzed in parallel with the samples. Minor residues only of some 2-4 ringed parent PAHs occurred, although the amount occurring was <10% of the amount found in the samples. All results were corrected for the blanks. The limits of detection (LOD) were calculated as three times the standard deviation of the values for the blanks or the background noise of these blanks. One standard reference material (SRM 1650b) was used as quality control (QC). The measured levels lie, for most part, within 30% of the certified levels (Figure S2).


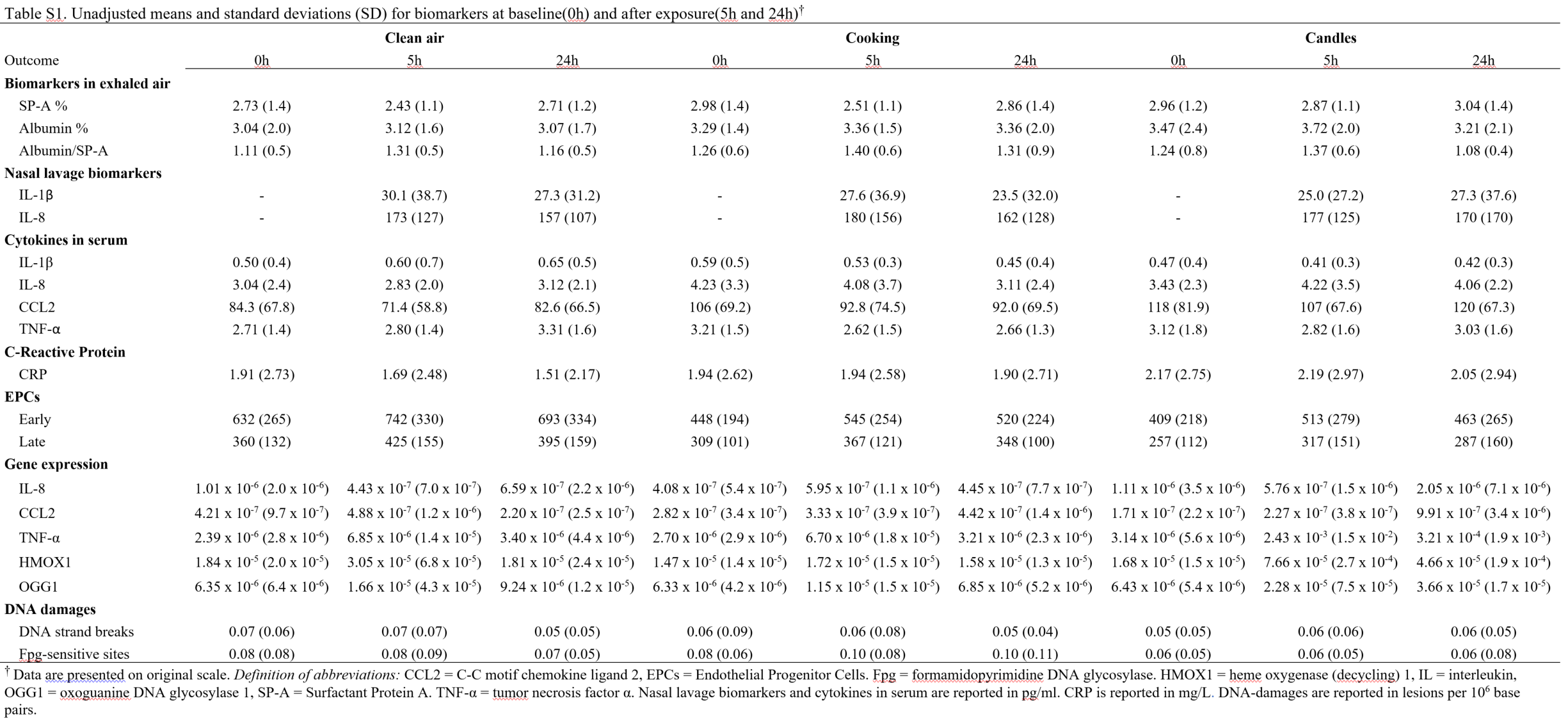


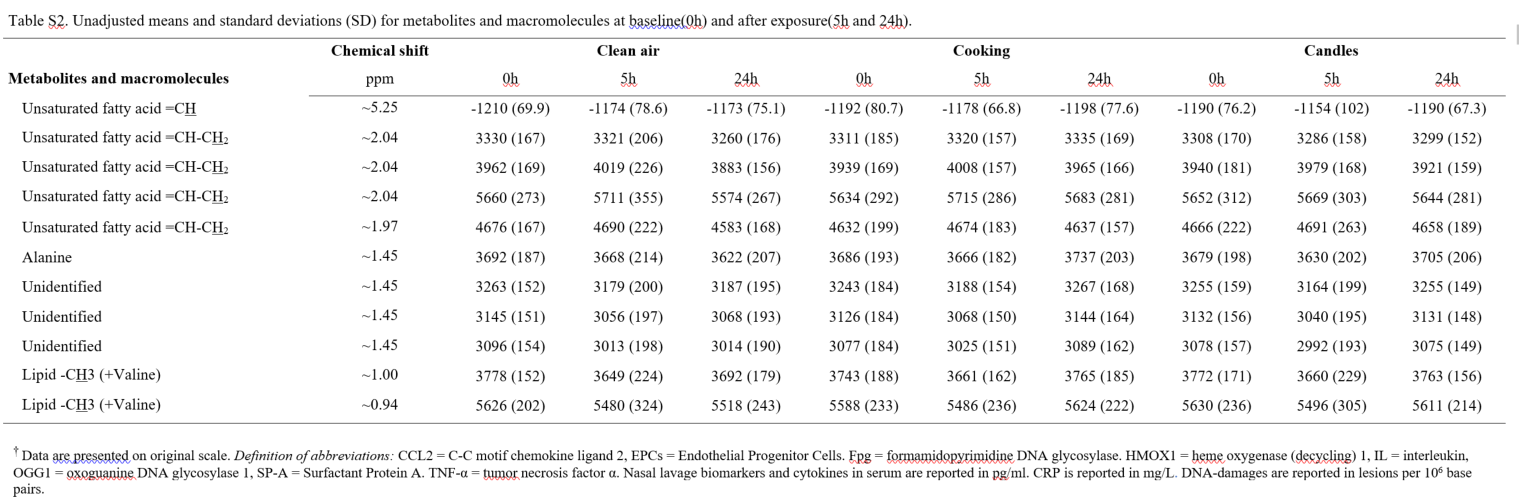


| Table S3. Mean change (0h, 5h, and 24 h) in biomarkers in exhaled air on days with cooking and candle exposure (clean air = reference) ^†^ | | | | | | | |
| --- | --- | --- | --- | --- | --- | --- | --- |
|  |  | **Cooking** | | | **Candles** | | |
| **Biomarkers** | | Coefficient | 95% CI | *p*-value | Coefficient | 95% CI | *p*-value |
|  | SP-A % | 0.23 | (-0.06; 0.51) | 0.125 | 0.33 | (0.04; 0.61) | 0.027^*^ |
|  | Albumin % | 0.28 | (-0.15; 0.72) | 0.202 | 0.36 | (-0.08; 0.79) | 0.109 |
|  | Albumin/SP-A | 0.11 | (-0.03; 0.25) | 0.124 | 0.04 | (-0.11; 0.18) | 0.627 |
| ^†^ Results are from linear mixed models with no interaction term. *Definition of abbreviations*: SP-A = Surfactant Protein-A. * The level of significance was assumed at *p* < 0.05. | | | | | | | |

| Table S4. Mean change (5h-24 h) in biomarkers in exhaled air following cooking and candle exposure (clean air = reference)^†^ | | | | | | | |
| --- | --- | --- | --- | --- | --- | --- | --- |
|  |  | **Cooking** | | | **Candles** | | |
| **Biomarkers** | | Coefficient | 95% CI | *p*-value | Coefficient | 95% CI | *p*-value |
|  | SP-A % | 0.18 | (-0.18; 0.54) | 0.322 | 0.40 | (0.04; 0.76) | 0.032^*^ |
|  | Albumin % | 0.32 | (-0.20; 0.84) | 0.228 | 0.35 | (-0.17; 0.87) | 0.184 |
|  | Albumin/SP-A | 0.10 | (-0.07; 0.27) | 0.243 | -0.02 | (-0.19; 0.15) | 0.821 |
| ^†^ Results are from linear mixed models with no interaction term and no adjustment for baseline values. SP-A and albumin are expressed as weight percent. *Definition of abbreviations*: SP-A = Surfactant Protein-A. * The level of significance was assumed at *p* < 0.05. | | | | | | | |

Table S5. Concentrations (average^a^ and range) of PM_2.5_associated 16 US EPA PAHs (ng/m^3^)

| **Measurement** | **Clean air**  **Mean Range** | | **Cooking**  **Mean Range** | | **Candles**  **Mean Range** | |
| --- | --- | --- | --- | --- | --- | --- |
| Number of sessions, N | 10 | | 11 | | 11 | |
| naphthalene | 0.37 | 0.073 - 0.63 | 0.18 | 0.050 - 0.25 | 0.89 | 0.57 - 1.7 |
| acenaphthylene | 0.0063 | <0.0018 - 0.011 | 0.012 | 0.0052 - 0.021 | 0.079 | 0.057 - 0.12 |
| acenaphthene | 0.018 | 0.0042 - 0.039 | 0.0094 | 0.0052 - 0.014 | 0.053 | 0.033 - 0.12 |
| fluorene | 0.060 | 0.011 - 0.10 | 0.059 | 0.021 - 0.12 | 0.40 | 0.25 - 0.80 |
| phenanthrene | 0.12 | 0.023 - 0.25 | 0.13 | 0.10 - 0.17 | 4.1 | 2.9 - 8.0 |
| anthracene | 0.015 | <0.0044 - 0.020 | 0.013 | <0.0044 - 0.024 | 0.25 | 0.15 - 0.59 |
| fluoranthene | 0.039 | 0.0076 - 0.080 | 0.11 | 0.087 - 0.19 | 1.3 | 0.97 - 3.4 |
| pyrene | 0.059 | 0.012 - 0.096 | 0.34 | 0.26 - 0.42 | 2.6 | 1.8 - 5.0 |
| benzo(a)anthracene | 0.0023 | <0.0017 - 0.0034 | 0.0079 | <0.0017 - 0.017 | 0.014 | <0.0017 - 0.051 |
| chrysene | 0.0093 | 0.0029 - 0.022 | 0.058 | 0.027 - 0.096 | 0.12 | 0.060 - 0.33 |
| benzo(b)fluoranthene | 0.067 | 0.015 - 0.13 | 0.13 | 0.064 - 0.18 | 0.15 | 0.31 - 0.11 |
| benzo(k)fluoranthene | 0.0058 | <0.0021 - 0.014 | 0.014 | 0.0032 - 0.032 | 0.037 | 0.0087 - 0.38 |
| benzo(a)pyrene | 0.010 | 0.0027 - 0.015 | 0.026 | 0.013 - 0.064 | 0.037 | 0.023 - 0.061 |
| indeno(1,2,3-c,d)pyrene | 0.0058 | <0.0020 - 0.011 | 0.012 | 0.0026 - 0.030 | 0.033 | 0.018 - 0.060 |
| dibenzo(a,h)anthracene | <0.0024 | <0.0024 | 0.0040 | <0.0024 - 0.0040 | 0.017 | <0.0024 - 0.030 |
| benzo(g,h,i)perylene | 0.016 | 0.0041 - 0.025 | 0.030 | 0.0087 - 0.21 | 0.065 | 0.027 - 0.13 |
| **SUM PAHs** | **0.70** | **0.16 - 1.3** | **1.1** | **0.88 - 1.6** | **10** | **7.8 - 21** |
| ^a^ The average value for each session is based on the following number of samples; clean air (n=15), cooking (n=22) and candles (n=16). | | | | | | |

**Figure S1**

**
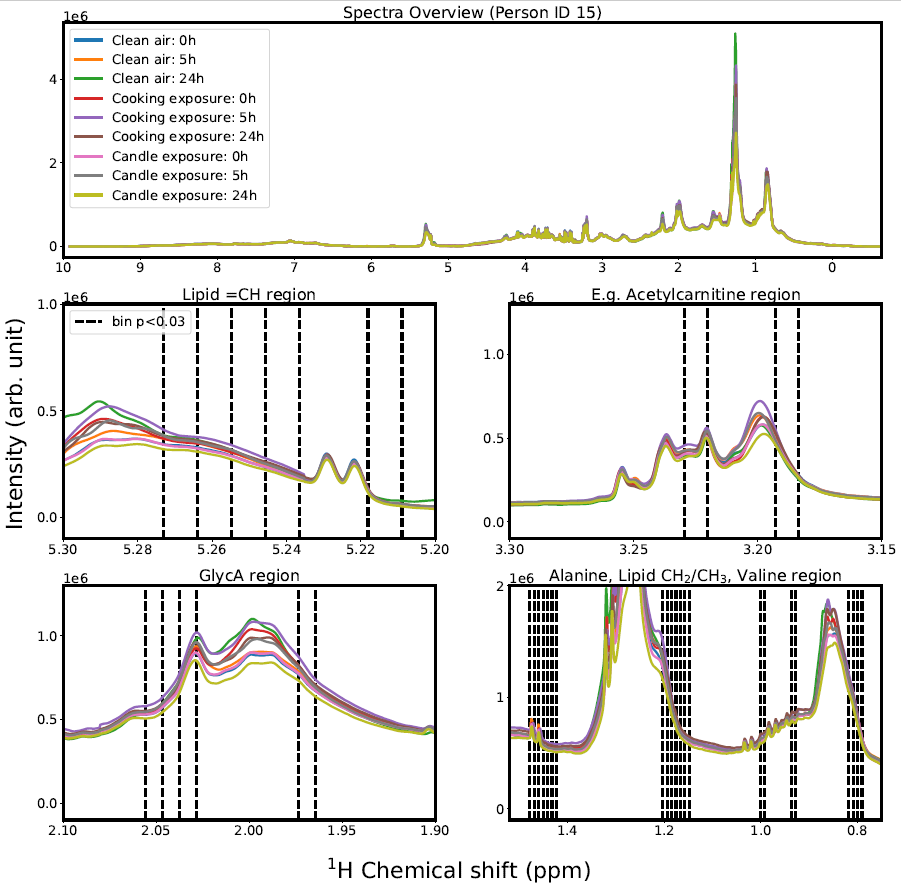
**

Figure S1. Spectra overview of example NMR dataset of person with ID 15. Interesting regions are shown in subfigures where significant changes of metabolites and macromolecules were observed between exposure to clean air and exposure to candle / cooking (exact bins lie between dotted black lines).

**Figure S2**

Figure S2. Results of three quality control (QC) samples (NIST SRM 1649b) compared to published certified values of 20 polycyclic aromatic compounds.
